# Supplementary material for: Structural basis for energy transfer in a huge diatom PSI-FCPI supercomplex
Source: Nat Commun. 2020 Oct 8;11:5081. doi: 10.1038/s41467-020-18867-x (PMC7545214; doi:10.1038/s41467-020-18867-x)
Supplement: Supplementary file 1 — Supplementary Information [file 41467_2020_18867_MOESM1_ESM.pdf]

Supplementary Information

Structural basis for energy transfer in a huge diatom PSI-FCPI supercomplex

Caizhe Xu, Xiong Pi, Yawen Huang, Guangye Han, Xiaobo Chen, Xiaochun Qin, Guoqiang Huang, Songhao Zhao, Yanyan Yang, Tingyun Kuang, Wenda Wang, Sen-Fang Sui, Jian-Ren Shen

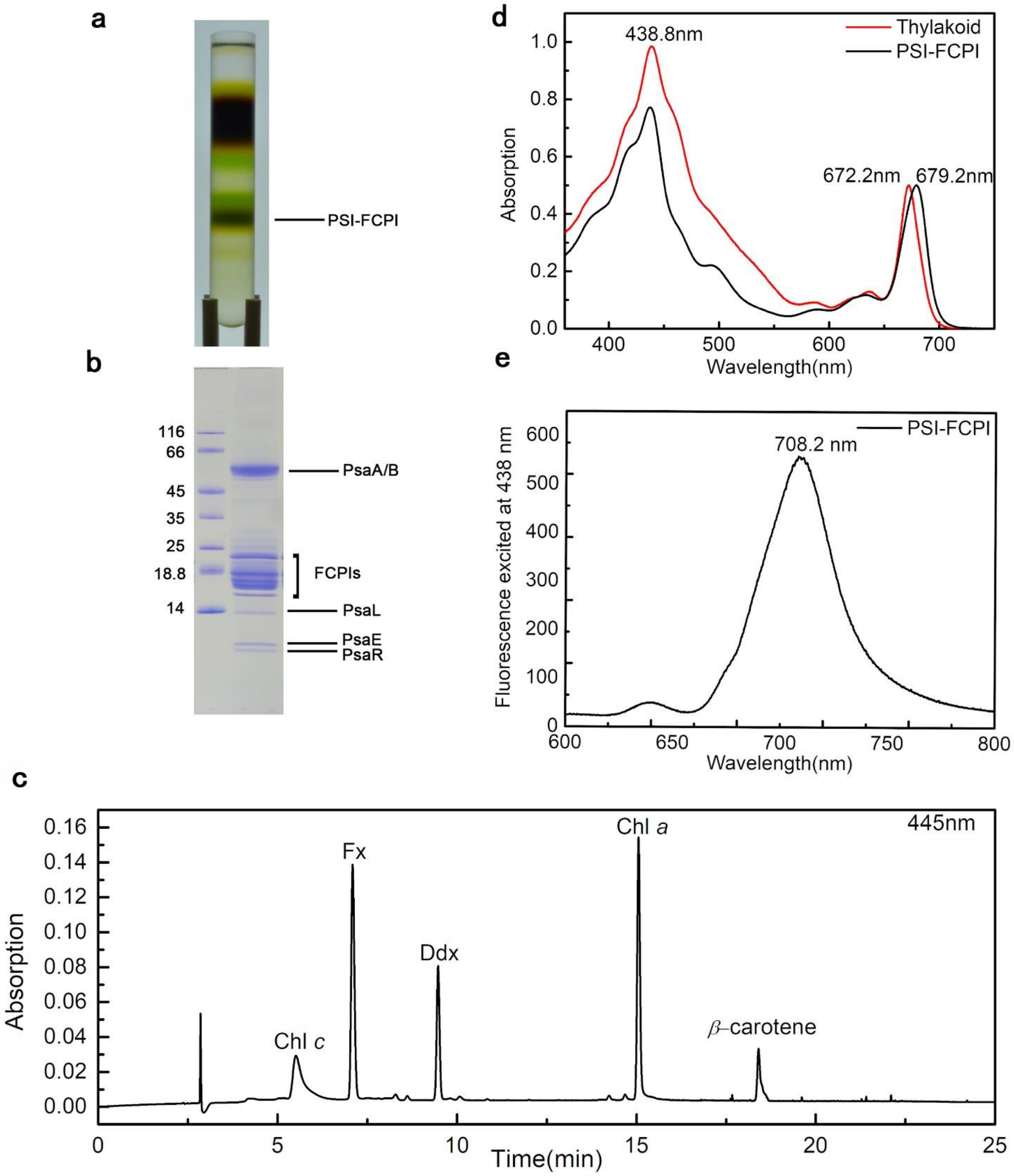

Supplementary Fig. 1. Purification and characterization of *C. gracilis* PSI-FCPI. a, Separation of the PSI-FCPI supercomplex by sucrose density gradient centrifugation. **b**, SDS-PAGE analysis of the PSI-FCPI supercomplex. **c**, Pigment analysis of PSI-FCPI by high performance liquid chromatography (HPLC), recorded at 445 nm. **d**, Absorption spectra of the thylakoid membrane and PSI-FCPI supercomplex. **e**, Fluorescence emission spectrum of PSI-FCPI at 77 K excited at 438 nm.

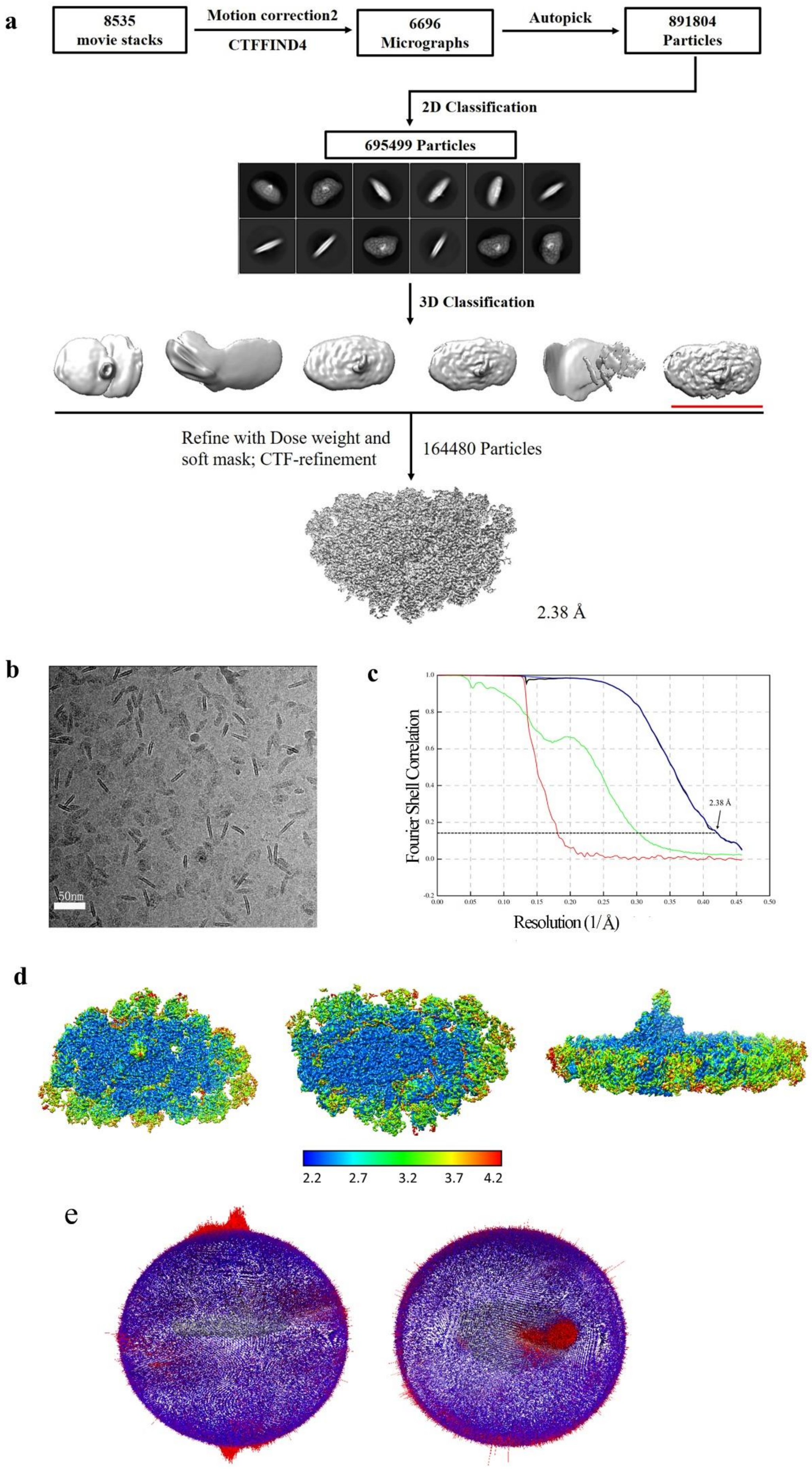
a
8535
movie stacks
Motion correction2
CTFFIND4
6696
Micrographs
Autopick
891804
Particles
2D Classification
695499 Particles
3D Classification
Refine with Dose weight and
soft mask; CTF-refinement
164480 Particles
2.38 Å
b
50nm
c
Fourier Shell Correlation
Resolution (1/Å)
2.38 Å
d
2.2
2.7
3.2
3.7
4.2
e

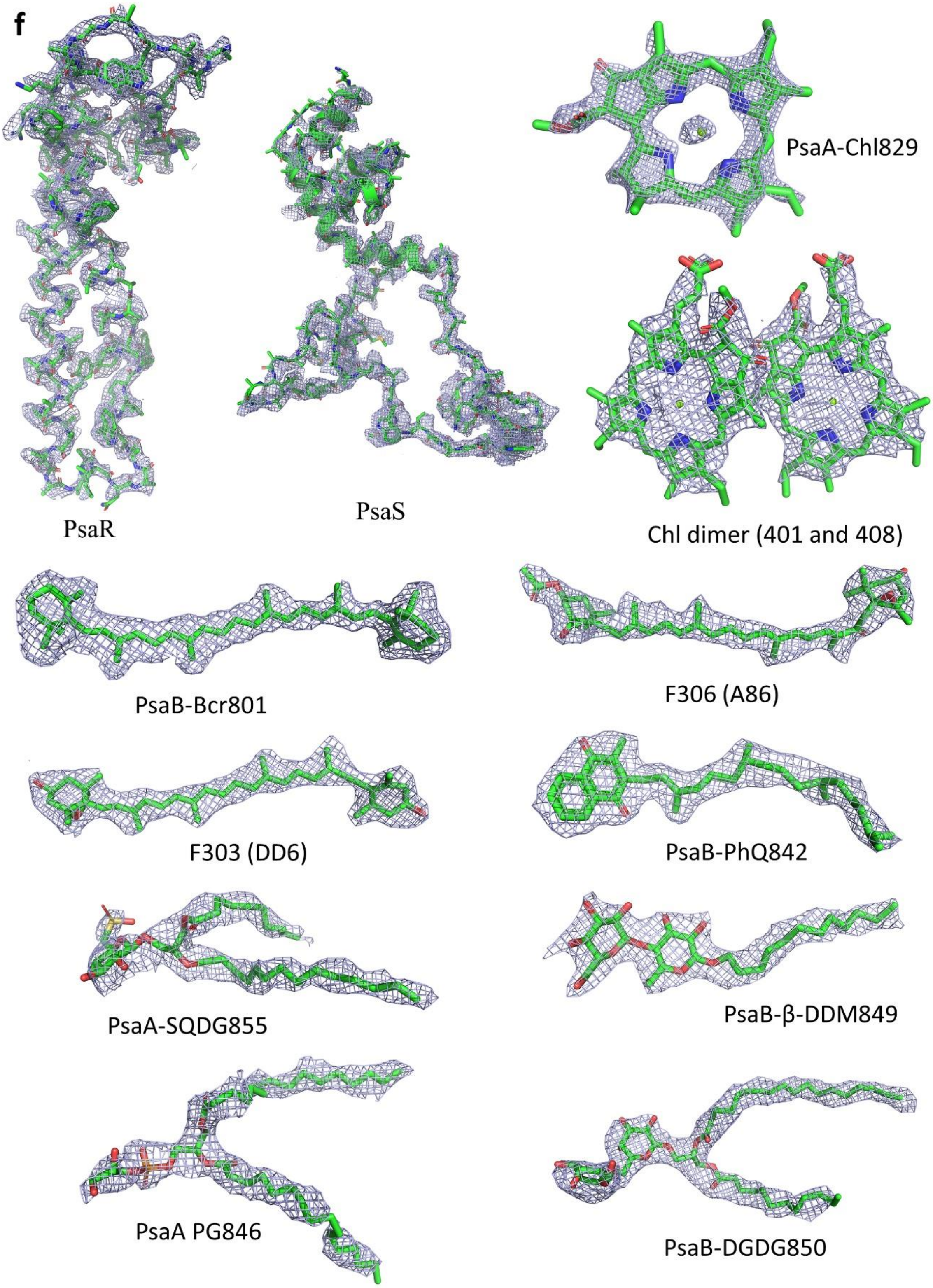

Supplementary Fig. 2. Single particle cryo-EM analysis of the diatom PSI-FCPI supercomplex. a, Flowchart for the cryo-EM data processing. **b,** A Cryo-EM image of the PSI-FCPI supercomplex. **c,** The gold standard Fourier shell correlation (FSC) curve for estimation of the resolution with the criterion of FSC 0.143. Black, corrected; blue, masked maps, green, unmasked maps; red, phase-randomized, masked maps. **d,** Full local resolution maps for the diatom PSI-FCPI supercomplex. Left, view from the lumenal side; middle, view from the stromal side; right, side view. **e,** Euler distribution plot of the density map. **f,** Cryo-EM maps for the two novel subunits PsaR, PsaS and some representative cofactors.

a

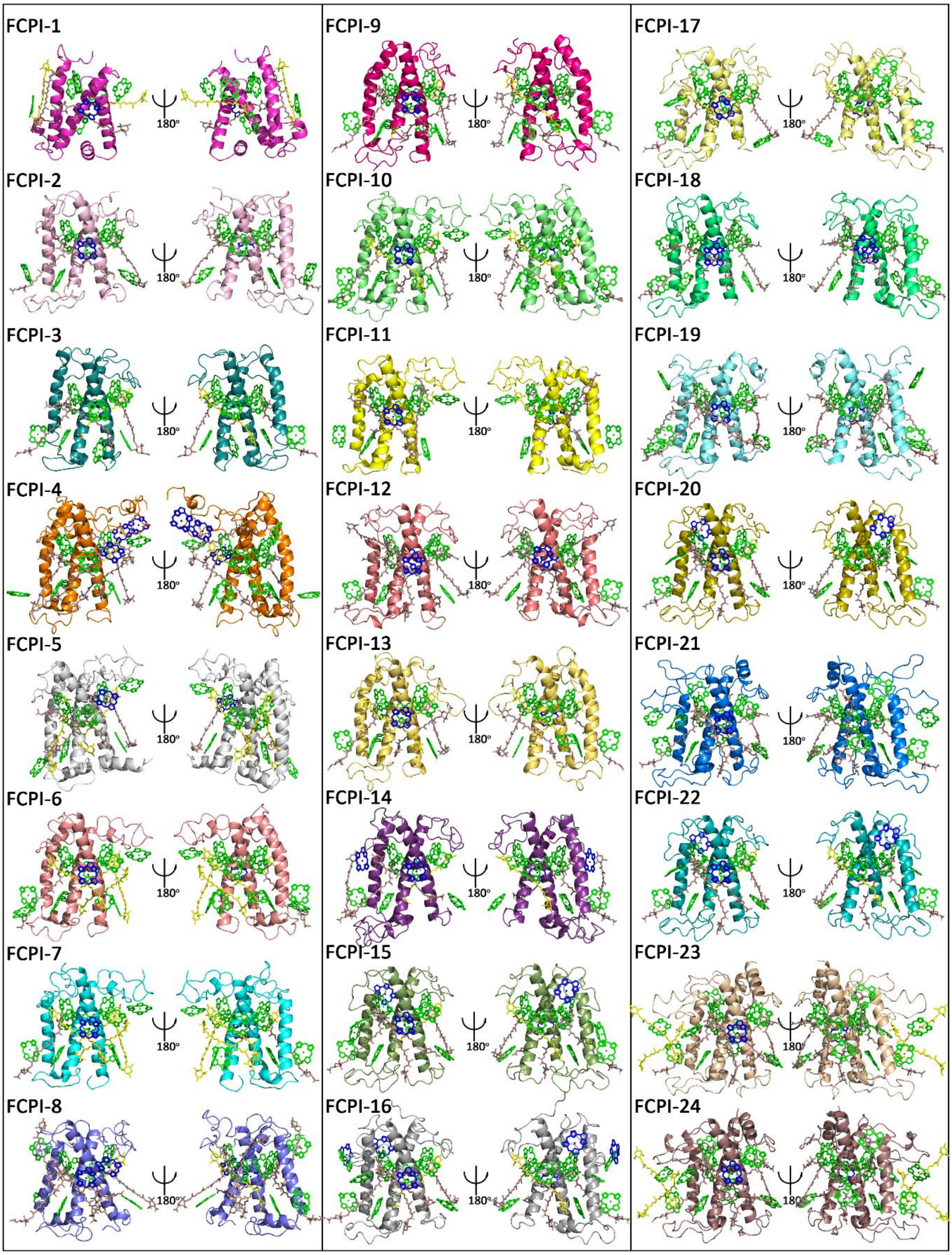

FCPI-1
FCPI-2
FCPI-3
FCPI-4
FCPI-5
FCPI-6
FCPI-7
FCPI-8
FCPI-9
FCPI-10
FCPI-11
FCPI-12
FCPI-13
FCPI-14
FCPI-15
FCPI-16
FCPI-17
FCPI-18
FCPI-19
FCPI-20
FCPI-21
FCPI-22
FCPI-23
FCPI-24
180°

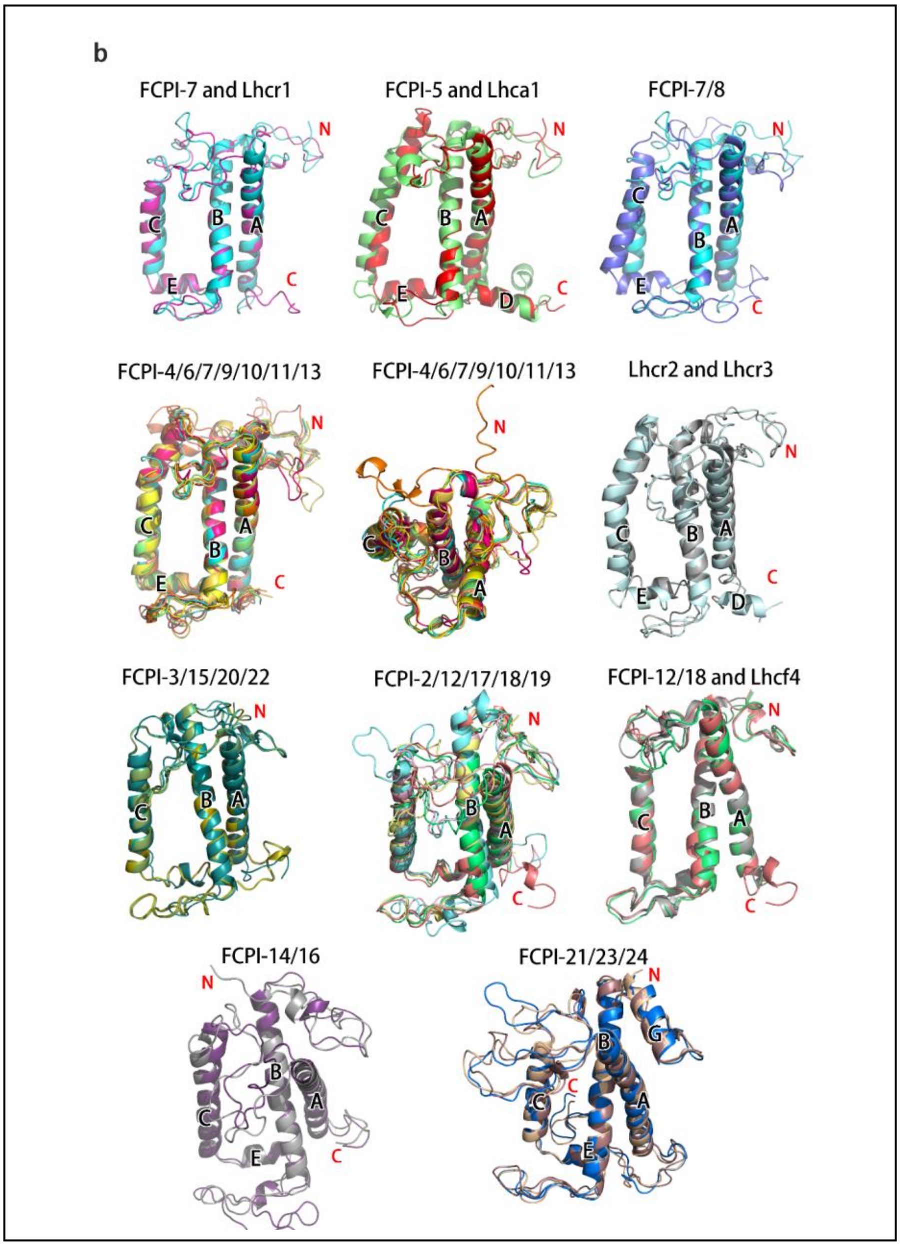

Supplementary Fig. 3. Structures of individual FCPIs and their comparisons with other Lhcr and Lhcf proteins. a, Structures of the 24 FCPIs. Chl *a*, Chl *c,* Ddx and Fx are shown in sticks with the phytol chains omitted and colored in green, blue, yellow and darksalmon, respectively. **b,** Comparisons of the structures among different FCPIs and between FCPIs and Lhcr1, Lhcr2, Lhcr3 from a red alga *C. merolae*[1], Lhc a1 from *Pisum sativum*[2], and Lhcf4 from *P. tricornutum*[3]. Based on the structures the 24 FCPIs are divided into five groups. Group I (FCPI-4/5/6/7/8/9/10/11/13) belongs to Lhcr1/2/3-type proteins. Their comparisons with Lhcr1/2/3 from red algae and Lhca1 from *P. sativum* are shown in the first and second row. Group II (FCPI-2/3/12/15/17/18/19/20/22) comparison with Lhf4 from *P. tricornutum* is shown in the third row, and group III (FCPI-14/16) and IV (FCPI-21/23/24) are shown in the fourth row. The unique FCPI-1 belongs to group V and was shown in Fig. 4b. The color code of FCPI subunits in supplementary Fig. 3b are the same as those in supplementary Fig. 3a, and Lhcr 1/2/3 of red algae are in magenta, aquamarine and gray colors while the Lhca1 of *P. sativum* is in green.

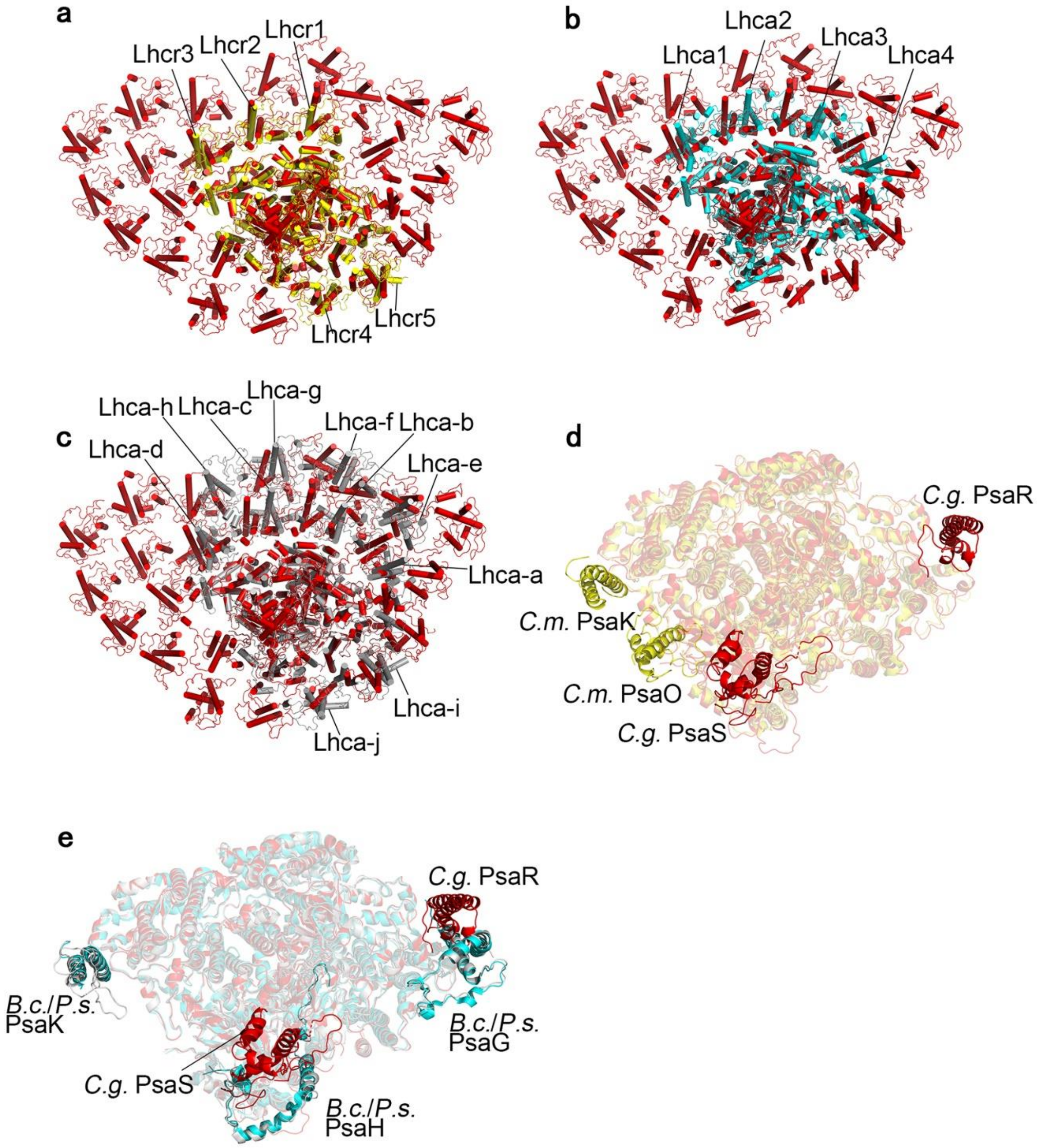

Supplementary Fig. 4. Comparison of the structures of *C. gracilis* PSI-FCPI with that of *C. merolae, B. corticulans* and *P. sativum*. a, b, c, Superposition of the diatom PSI-FCPI with PSI-LHCI of a red alga *C. merolae* **(a)**, a higher plant *P. sativum* **(b)**, and a green alga *B. corticulans* **(c)**, showing the differences in the type and number of antennas among the three PSI-LHCI. **d**, **e,** Superposition of the PSI core structures between the diatom and other three species. All panels are viewed from the stromal side. Color codes used**:** Red, *C. gracilis* PSI-FCPI; yellow, *C. merolae* PSI-LHCI; grey, *B. corticulans* PSI-LHCI; cyan, pea PSI-LHCI.

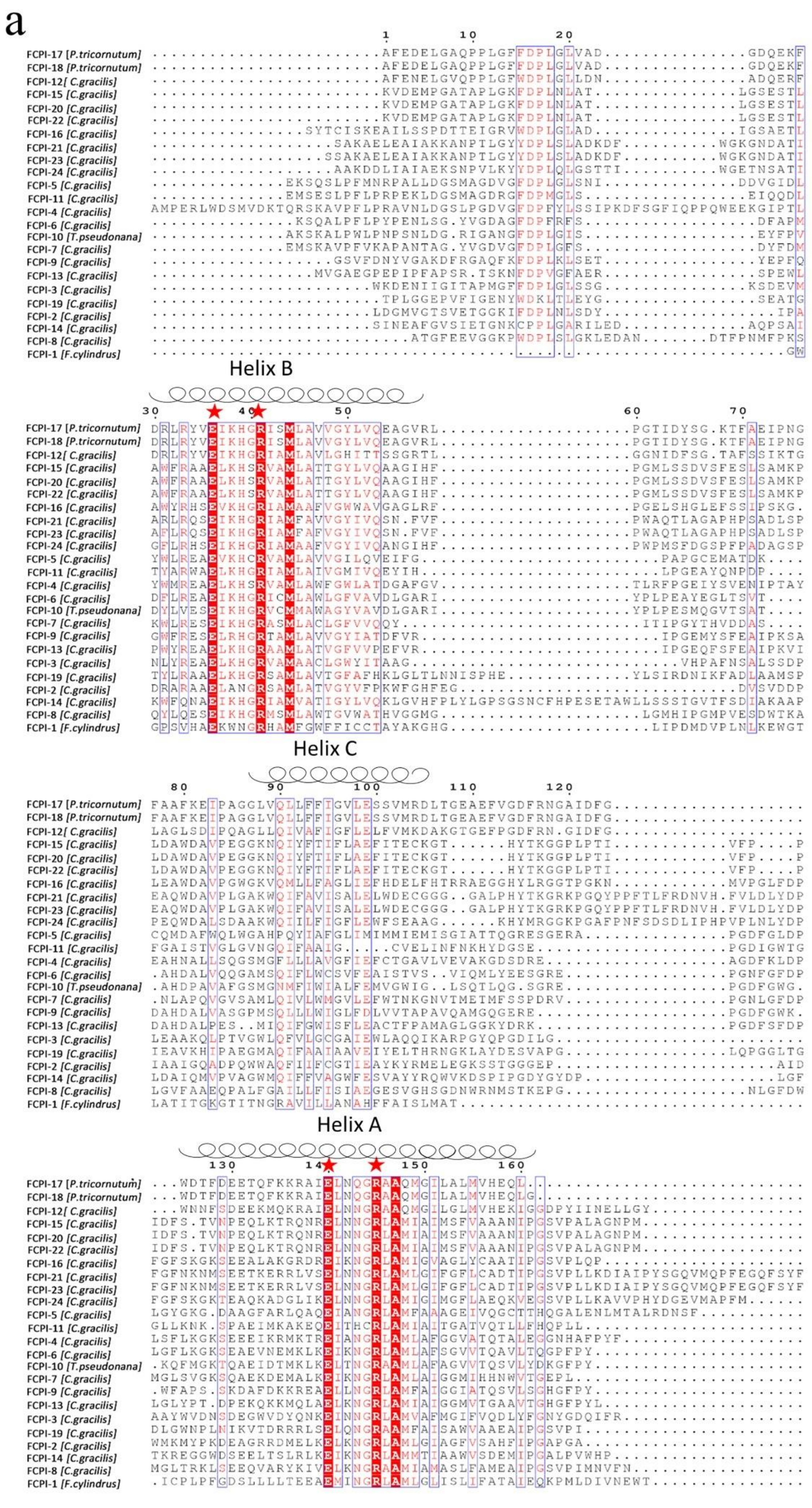
a
Helix B
Helix C
Helix A

b

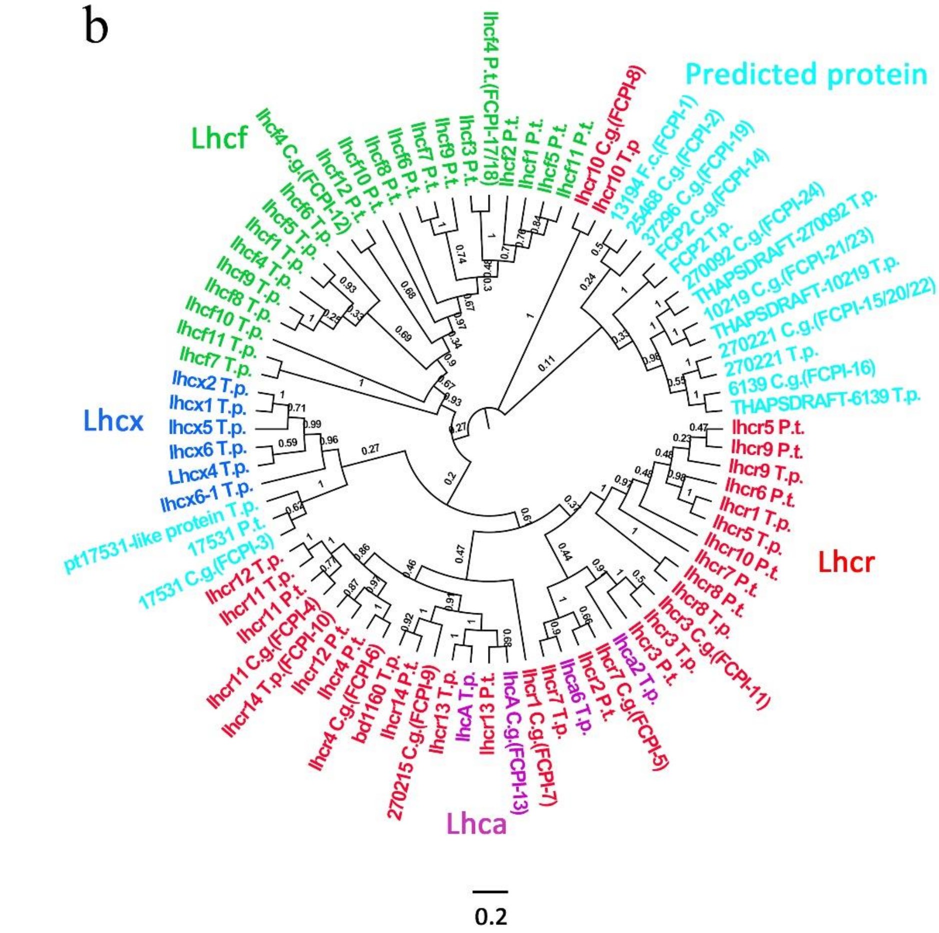
Lhcf
Lhcx
Predicted protein
Lhcr
Lhca
lhcf4 P.t.(FCPI-17/18)
lhcf3 P.t.
lhcf2 P.t.
lhcf1 P.t.
lhcf5 P.t.
lhcf11 P.t.
lhcf9 P.t.
lhcf7 P.t.
lhcf6 P.t.
lhcf8 P.t.
lhcf10 P.t.
lhcf12 P.t.
lhcf4 C.g.(FCPI-12)
lhcf6 T.p.
lhcf5 T.p.
lhcf1 T.p.
lhcf4 T.p.
lhcf9 T.p.
lhcf8 T.p.
lhcf10 T.p.
lhcf11 T.p.
lhcf7 T.p.
lhcx2 T.p.
lhcx1 T.p.
lhcx5 T.p.
lhcx6 T.p.
Lhcx4 T.p.
lhcx6-1 T.p.
pt17531-like protein T.p.
17531 P.t.
17531 C.g.(FCPI-3)
lhcr10 C.g.(FCPI-8)
lhcr10 T.p
13194 F.c.(FCPI-1)
25468 C.g.(FCPI-2)
37296 C.g.(FCPI-19)
FCP2 C.g.(FCPI-14)
FCP2 T.p.
270092 C.g.(FCPI-24)
THAPSDRAFT-270092 T.p.
10219 C.g.(FCPI-21/23)
THAPSDRAFT-10219 T.p.
270221 C.g.(FCPI-15/20/22)
270221 T.p.
6139 C.g.(FCPI-16)
THAPSDRAFT-6139 T.p.
lhcr5 P.t.
lhcr9 P.t.
lhcr9 T.p.
lhcr6 P.t.
lhcr1 T.p.
lhcr5 T.p.
lhcr10 P.t.
lhcr7 P.t.
lhcr8 T.p.
lhcr8 P.t.
lhcr3 C.g.(FCPI-11)
lhcr3 T.p.
lhcr3 P.t.
lhca2 T.p.
lhcr7 C.g.(FCPI-5)
lhcr2 P.t.
lhca6 T.p.
lhcr7 T.p.
lhcr1 C.g.(FCPI-7)
lhcA C.g.(FCPI-13)
lhcr13 P.t.
lhcA T.p.
lhcr13 T.p.
270215 C.g.(FCPI-9)
lhcr14 P.t.
bd1160 T.p.
lhcr4 C.g.(FCPI-6)
lhcr4 P.t.
lhcr12 P.t.
lhcr14 T.p.(FCPI-10)
lhcr11 C.g.(FCPI-4)
lhcr11 P.t.
lhcr11 T.p.
lhcr12 T.p.
0.2

c

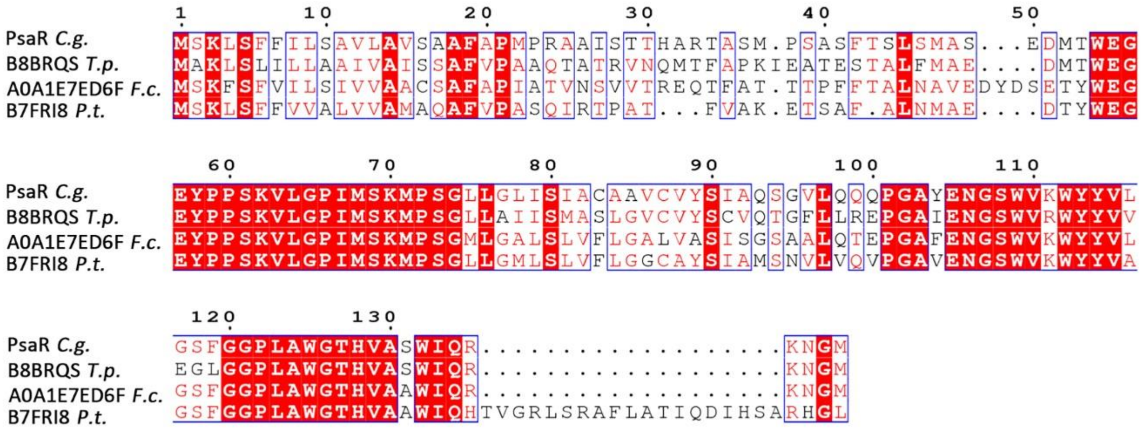
PsaR C.g. MSKLSFFILSAVLAVSAAFAPMPRAAISTTHARTASM.PSASFTSLSMAS...EDMTWEG
B8BRQS T.p. MAKLSLILLAAIVAISSAFVPAAQTATRVNQMTFAPKIEATESTALFMAE....DMTWEG
A0A1E7ED6F F.c. MSKFSFVILSIVVAACSAFAPIATVNSVVTREQTFAT.TTPFFTALNAVEDYDSETYWEG
B7FRI8 P.t. MSKLSFFVVALVVAMAQAFVPASQIRTPAT...FVAK.ETSAF.ALNMAE....DTYWEG
PsaR C.g. EYPPSKVLGPIMSKMPSGLLGLISIACAAVCVYSIAQSGVLQQQPGAYENGSWVKWYYVL
B8BRQS T.p. EYPPSKVLGPIMSKMPSGLLAIISMASLGVCVYSCVQTGFLLREPGAIENGSWVRWYYVV
A0A1E7ED6F F.c. EYPPSKVLGPIMSKMPSGMLGALSLVFLGALVASISGSAALQTEPGAFENGSWVKWYYVL
B7FRI8 P.t. EYPPSKVLGPIMSKMPSGLLGMLSLVFLGGCAYSIAMSNVLVQVPGAVENGSWVKWYYVA
PsaR C.g. GSFGGPLAWGTHVASWIQR....................KNGM
B8BRQS T.p. EGLGGPLAWGTHVASWIQR....................KNGM
A0A1E7ED6F F.c. GSFGGPLAWGTHVAAWIQR....................KNGM
B7FRI8 P.t. GSFGGPLAWGTHVAAWIQHTVGRLSRAFLATIQDIHSARHGL

d

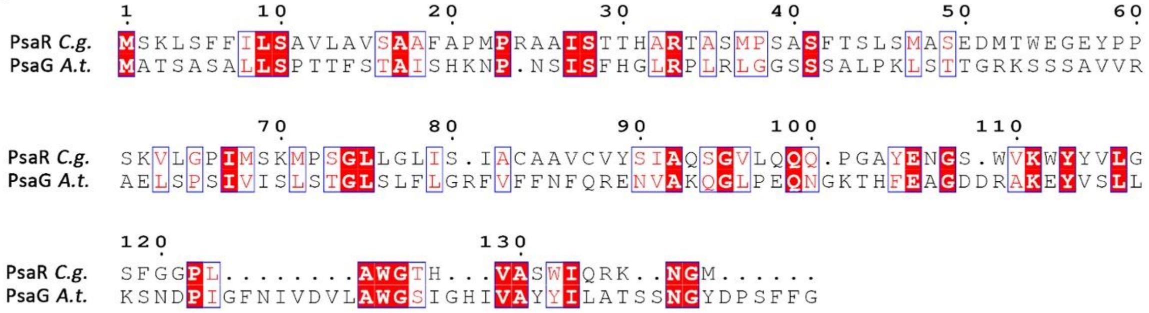
PsaR C.g. MSKLSFFILSAVLAVSAAFAPMPRAAISTTHARTASMPSASFTSLSMASEDMTWEGEYPP
PsaG A.t. MATSASALLSPTTFSTAISHKNP.NSISFHGLRPLRLGGSSSALPKLSTTGRKSSSAVVR
PsaR C.g. SKVLGPIMSKMPSGLLGLIS.IACAAVCVYSIAQSGVLQQQ.PGAYENGS.WVKWYYVLG
PsaG A.t. AELSPSIVISLSTGLSLFLGRFVFFNFQRENVAKQGLPEQNGKTHFEAGDDRAKEYVSLL
PsaR C.g. SFGGPL........AWGTH...VASWIQRK..NGM......
PsaG A.t. KSNDPIGFNIVDVLAWGSIGHIVAYYILATSSNGYDPSFFG

Supplementary Fig. 5. Phylogenetic analysis and amino acid sequences alignment of FCPIs. a, Alignment of FCPIs suquencess. The relationship between the numbering of FCPI and Uniprot ID is shown in supplementary Table 1. **b,** Phylogenetic tree of FCPs in diatoms. **c**, Sequence alignment of PsaR from four species of diatoms. **d**, Comparison of the sequences of PsaR from *C. gracilis* and PsaG from *P. sativum*. Sequence alignment and phylogentic analysis were performed using MEGA X[4] and Phylogeny.fr[5]. Residues painted with red represent the same amino acids, and residues within blue frames represent similar amino acids. Evolutionary history was analyzed based on the Neighbor-Joining method[6], and the optimal tree with a sum of branch length = 16.47780753 is shown. The percentage of replicate trees in which the associated taxa are clustered together in the bootstrap test (1000 replicates) are shown next to the branches[7]. The tree is drawn in scale, with branch lengths in the same units as those of the evolutionary distances used to infer the phylogenetic tree. The evolutionary distances were computed using the Poisson correction method[8], and are in the unit of the number of amino acid substitutions per site. All ambiguous positions were removed for each sequence pair (pairwise deletion option). There was a total of 414 positions in the final dataset. The phylogenetic tree is optimized using fig tree1.4.4[9].

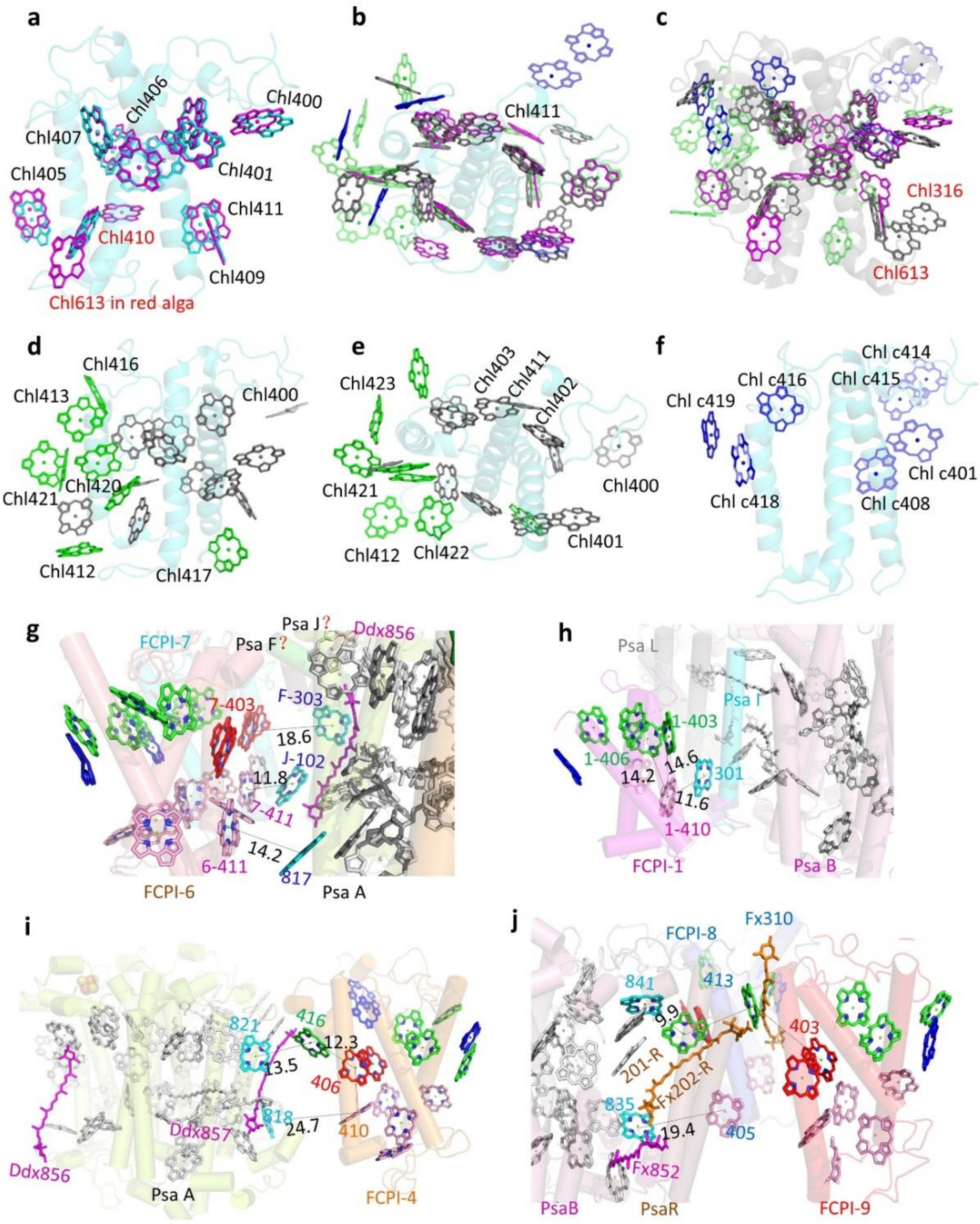
a
Chl406
Chl400
Chl407
Chl401
Chl405
Chl411
Chl410
Chl409
Chl613 in red alga
b
Chl411
c
Chl316
Chl613
d
Chl416
Chl413
Chl400
Chl420
Chl421
Chl412
Chl417
e
Chl423
Chl403
Chl411
Chl402
Chl421
Chl400
Chl412
Chl422
Chl401
f
Chl c414
Chl c416
Chl c415
Chl c419
Chl c401
Chl c418
Chl c408
g
FCPI-7
Psa J?
Psa F?
Ddx856
7-403
F-303
18.6
J-102
11.8
7-411
6-411
14.2
817
FCPI-6
Psa A
h
Psa L
Psa I
1-403
1-406
14.2
14.6
301
11.6
1-410
FCPI-1
Psa B
i
821
416
12.3
13.5
406
818
24.7
410
Ddx857
Ddx856
Psa A
FCPI-4
j
Fx310
FCPI-8
841
413
9.9
403
201-R
Fx202-R
835
19.4
405
Fx852
PsaB
PsaR
FCPI-9

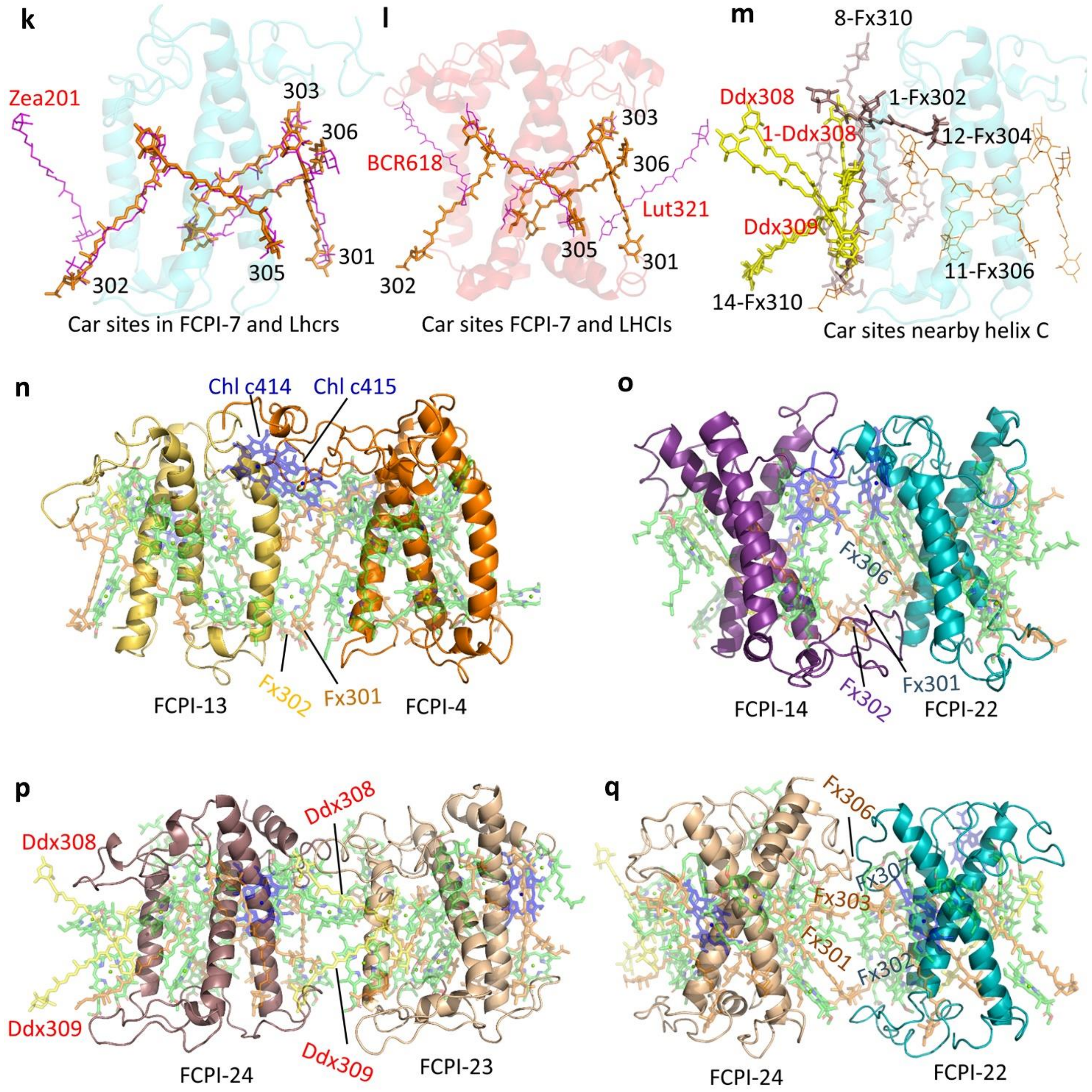

Supplementary Fig. 6. Pigment-binding sites in diatom FCPIs and comparisons with other LHCIs, and EET pathways from antennas to the PSI core. a, Comparison of Chl sites in FCPI-7 (cyan) and three Lhcrs (magenta) from a red alga[1]. Chl613 in red algal Lhcr2 are labeled in red color. Chl410 in FCPI-4/6/9/10/13 and FCPII, but missing in FCPI-7 are also shown in red color. **b**, **c,** Twenty-four Chl sites of FCPIs and their comparisons with Chl sites in Lhcrs and Lhcas. Chls *a* and *c* in FCPIs are colored in green and blue, respectively. Chls in Lhcrs and Lhcas are shown in magenta and gray respectively. Two sites (Chl613 and Chl316) bound to the C-terminal helix D of Lhcas are label in red color. **d, e,** Chl *a* sites among 24 Chls in FCPIs from side view and top view from stroma, respectively. Twelve Chls *a* (400-411) are depicted in gray and others are depicted in green. **f,** Chl *c* sites identified in FCPIs. **g,** EET from FCPI-6 and FCPI-7 to the PSI core mediated by PsaF and PsaJ. **h,** EET from FCPI-1 to the PSI core mediated by PsaI. **i,** EET from FCPI-G to PsaA. **j,** EET from FCPI-F and FCPI-9 to the PSI core mediated by PsaR. Chl a201 and Fx 202 of PsaR are labeled in brown color. In panels **g, h, i** and **j,** Chls in PSI core are shown in gray, and the Chls associated with EET are colored in cyan. The Chls *a* of FCPIs located at the stromal and luminal sides are colored in green and pink respectively. Special Chl *a*-*a* pair are colored in red and Chls *c* are

colored in blue. Two Fx and two Ddx in the PSI core are labeled. **k,** Five Fxs (stick, orange) in FCPI-7 identical to those in red algal Lhcrs (ribbon, magenta). An additional Zea201 was found in Lhcr3. **l,** Comparison of Fxs in FCPI-7 with those in LHCIs (ribbon, magenta). Two central Car sites of LHCIs are conserved, whereas Lut321 and Bcr618 are lost and additional Fxs appeared in FCPIs. **m,** Highlight of Car-binding sites near helix C of FCPI. **n, o, p, q,** Pigment-pigment interactions among FCPI antennas. The Cars involved in interactions among FCPI antennas are labeled in the same color of the FCPI they are associated (FCPI-4, 12, 14, 22, 23, 24).

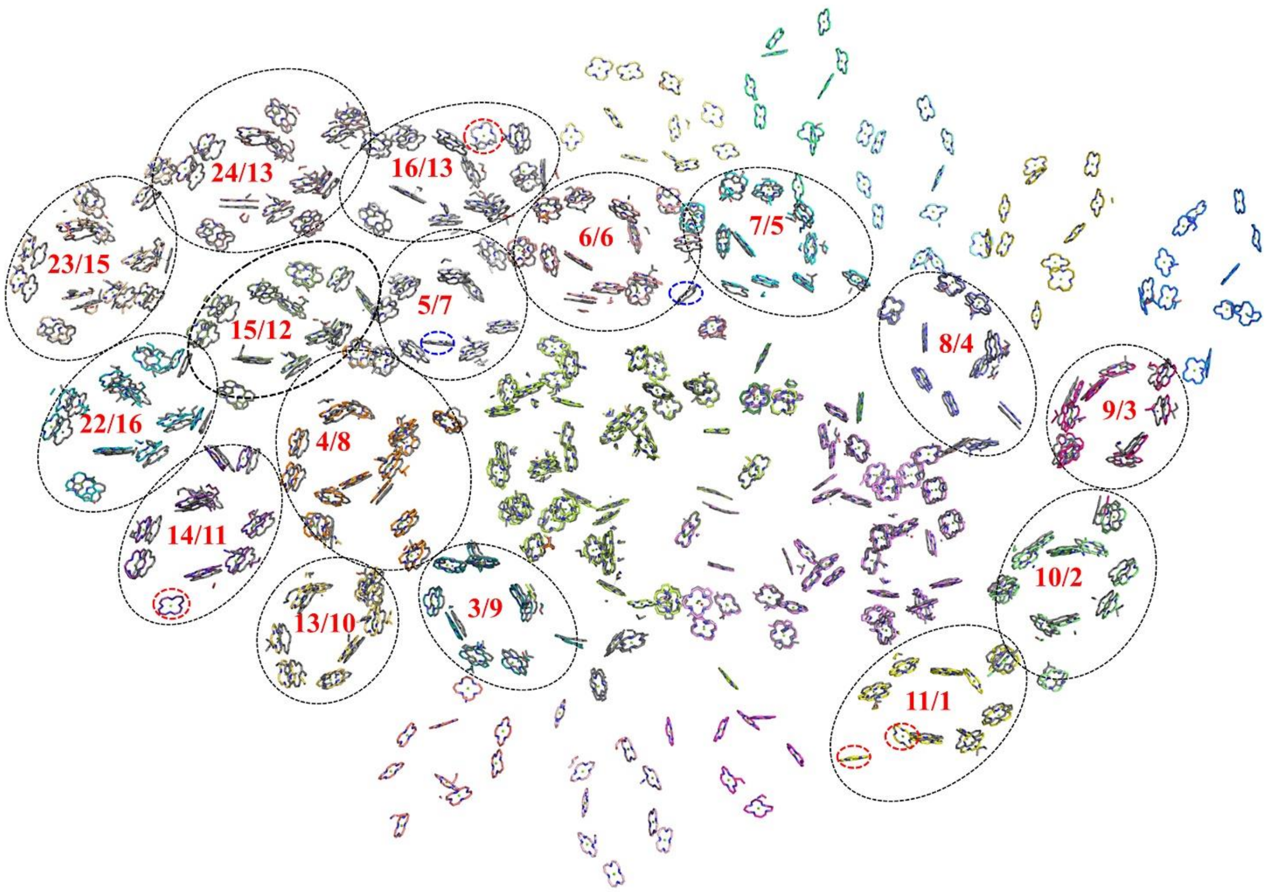

Supplementary Fig. 7. Superposition of the Chls assigned in the present structure with those in the Nagao et al.'s (2020)[10] structure. The Chls in the current structure are differently colored, and those of the Nagao et al.'s structure are in grey. Numbers represent the FCPI numbers in the present/Nagao et al.' structure. Only FCPI found in both structures are cycled by black dashed lines. Chls found in the current structure but absent in the Nagao et al.'s structure were cycled by red broken lines, whereas Chls present in Nagao et al.'s structure but absent in the current structure were cycled by blue broken lines.

Supplementary Table 1. Amino acid sequences and cofactors constructed in *C. gracilis* PSI-FCPI structure.

Subunits	Chain	Source (UniProt ID)	Residues	Chls	Cars	Lipids and ligands
PsaA	a	TM *C. gracilis* [a]	16-757	45 a	4 Bcr, 2 Ddx	1 SQDG, 2 MGDG 3 PG, 1 PQ, 1 Fe_4S_4
PsaB	b	TM *C. gracilis*<br>*P. sativum* (P05311)	2-723<br>724-733	40 a	7 Bcr, 1 Fx	1 DPPG, 1 DGDG, 1 PQ
PsaC	c	*P. sativum* (P10793)	2-81			2 Fe_4S_4
PsaD	d	*P. sativum* (A5Z2K3)[b]	79-210			
PsaE	e	*P. sativum* (E1C9K6)[c]	2-64			
PsaF	f	*P. sativum* (P12355)[d]	77-238	3 a	1 Bcr	1 MGDG, 1 PG
PsaI	i	*P. sativum* (P17227)	2-34	1 a	1 Bcr	
PsaJ	j	*P. vulgaris* (A4GGC6)	1-41	1 a	1 Bcr	
PsaL	l	TM *C. gracilis* [e]	4-147	3 a	3 Bcr	
PsaM	m	*C. merolae* (Q85G73)	2-29		1 Bcr	
PsaR	h	TM *C. gracilis*	51-139	1 a	1 Fx	
PsaS	g	polyA	17-150			
FCPI-1: Fc13194	B	*F. cylindrus* (A0A1E7F4Y9)[f]	60-218	6 a, 1 c	2 Ddx, 2 Fx	
FCPI-2: Cg25468	U	TM *C. gracilis*	1-156	8 a, 1 c	4 Fx	
FCPI-3: Cg17531	I	TM *C. gracilis*	34-194	10 a	1 Ddx, 3 Fx	
FCPI-4: Cglhcr11	G	TM *C. gracilis*[g]	32-245	12 a, 3 c	1 Ddx, 6 Fx	1 DGDG
FCPI-5: Cglhcr7	E	TM *C. gracilis*	35-222	9 a, 1 c	3 Ddx, 3 Fx	1 MGDG, 1 PG
FCPI-6: Cglhcr4	D	TM *C. gracilis*	35-206	12 a, 1 c	4 Ddx, 1 Fx	
FCPI-7: CgLhcr1	A	TM *C. gracilis*	35-202	10 a, 1 c	4 Ddx, 2 Fx	1 SQDG
FCPI-8: Cglhcr10	F	TM *C. gracilis*	36-215	8 a, 2 c	1 Ddx, 7 Fx	1 PG
FCPI-9: Cg270215	J	TM *C. gracilis*	33-199	10 a, 1 c	1 Ddx, 4 Fx	
FCPI-10: TpLhcr14	H	*T. pseudonana* (B8C0K4)	33-202	12a, 1 c	2 Ddx, 3 Fx	
FCPI-11: Cglhcr3	C	TM *C. gracilis*	35-197	9 a, 1 c	1 Ddx, 3 Fx	1 DGDG
FCPI-12: Cglhcf4	T	TM *C. gracilis*	1-172	7 a, 2 c	7 Fx	
FCPI-13: CglhcA	K	TM *C. gracilis*	38-205	9 a, 1 c	2 Ddx, 3 Fx	
FCPI-14: CgFCP2	L	TM *C. gracilis*	34-229	8 a, 2 c	1 Ddx, 3 Fx	
FCPI-15: Cg270221	P	TM *C. gracilis*	32-204	10 a, 2 c	1 Ddx, 4 Fx	
FCPI-16: Cg6139	M	TM *C. gracilis*	57-243	10 a, 3 c	1 Ddx, 4 Fx	1 MGDG
FCPI-17: PtLhcf4	W	*P. tricornutum* (B7FRW2)	1-161	1 0a, 1 c	2 Ddx, 2 Fx	
FCPI-18: PtLhcf4	X	*P. tricornutum* (B7FRW2)	1-161	8 a, 2 c	5 Fx	
FCPI-19: Cg37296	V	*TM C. gracilis*	1-179	10 a, 1 c	6 Fx	
FCPI-20: Cg270221	O	TM *C. gracilis*	32-204	9 a, 2 c	1 Ddx, 4 Fx	2 PG
FCPI-21: Cg10219	N	TM *C. gracilis*	33-251	12 a, 1 c	6 Fx	
FCPI-22: Cg270221	Q	TM *C. gracilis*	32-204	9 a, 2 c	1 Ddx, 4 Fx	1 PG
FCPI-23: Cg10219	S	TM *C. gracilis*	32-251	12 a, 1 c	2 Ddx, 7 Fx	
FCPI-24: Cg270092	R	TM *C. gracilis*	36-246	12 a, 1 c	2 Ddx, 7 Fx	
Others	Y Z					11 MGDG, 12 PG
PSI-FCPI				326 a, 34 c	18 Bcr, 35 Ddx, 102 Fx	2 SQDG, 3 DGDG, 16 MGDG, 22 PG, 2 PQ, 3 Fe_4S_4

TM *C. gracilis*: Transcriptome of *C. gracilis*; DGDG, digalactosyldiacyl glycerol; MGDG, monogalactosyldiacyl glycerol; PG, phosphatidyl glycerol; SQDG, sulfoquinovosyldiacyl glycerol. a: The amino acid sequence of PsaA is composed of three transcriptional sequences. b: The L90 and F99 change to W90 and K99 in A5Z2K3. c: The V61 changes to Q61 in E1C9K6. d: PolyA is used in amino acid 228-238 of PsaF. e: Residues 146-147 of PsaL were used sequences of *P. sativum* (E1C9L1). f: The residues 153-179 were deleted in A0A1E7F4Y9. g: The L154 change to V154 in FCPI-4.

Supplementary Table 2. Pigment-binding sites in the 24 FCPI antennas.

Sites - r	Sites - d	FCPI - 1	FCPI - 2	FCPI - 3	FCPI - 4	FCPI - 5	FCPI - 6	FCPI - 7	FCPI - 8	FCPI - 9	FCPI - 10	FCPI - 11	FCPI - 12	FCPI - 13	FCPI - 14	FCPI - 15	FCPI - 16	FCPI - 17	FCPI - 18	FCPI - 19	FCPI - 20	FCPI - 21	FCPI - 22	FCPI - 23	FCPI - 24
601	400				Chl *a*	Chl *a*	Chl *a*	Chl *a*			Chl *a*	Chl *a*													
609	401		Chl *a*	Chl *a*	Chl *c*	Chl *c*	Chl *a*	Chl *a*	Chl *c*	Chl *a*	Chl *a*	Chl *a*	Chl *a*	Chl *a*	Chl *a*	Chl *a*	Chl *a*	Chl *a*	Chl *a*	Chl *a*	Chl *a*	Chl *a*	Chl *a*	Chl *a*	Chl *a*
602	402	Chl *a*	Chl *a*	Chl *a*	Chl *a*	Chl *a*	Chl *a*	Chl *a*	Chl *a*	Chl *a*	Chl *a*	Chl *a*	Chl *a*	Chl *a*	Chl *a*	Chl *a*	Chl *a*	Chl *a*	Chl *a*	Chl *a*	Chl *a*	Chl *a*	Chl *a*	Chl *a*	Chl *a*
603	403	Chl *a*	Chl *a*	Chl *a*	Chl *a*	Chl *a*	Chl *a*	Chl *a*	Chl *a*	Chl *a*	Chl *a*	Chl *a*	Chl *c*	Chl *a*	Chl *a*	Chl *a*	Chl *a*	Chl *a*	Chl *c*	Chl *a*	Chl *a*	Chl *a*	Chl *a*	Chl *a*	Chl *a*
604	404		Chl *a*	Chl *a*	Chl *a*	Chl *a*	Chl *a*	Chl *a*	Chl *a*	Chl *a*	Chl *a*	Chl *a*	Chl *a*	Chl *a*	Chl *a*	Chl *a*	Chl *a*	Chl *a*	Chl *a*	Chl *a*	Chl *a*	Chl *a*	Chl *a*	Chl *a*	Chl *a*
605	405	Chl *a*	Chl *a*	Chl *a*			Chl *a*	Chl *a*	Chl *a*	Chl *a*	Chl *a*	Chl *a*	Chl *a*	Chl *a*	Chl *a*	Chl *a*	Chl *a*	Chl *a*	Chl *a*	Chl *a*	Chl *a*	Chl *a*	Chl *a*	Chl *a*	Chl *a*
607	406	Chl *a*	Chl *a*	Chl *a*	Chl *a*	Chl *a*	Chl *a*	Chl *a*	Chl *a*	Chl *a*	Chl *a*	Chl *a*	Chl *a*	Chl *a*	Chl *a*	Chl *a*	Chl *a*	Chl *a*	Chl *a*	Chl *a*	Chl *a*	Chl *a*	Chl *a*	Chl *a*	Chl *a*
608	407	Chl *a*	Chl *a*	Chl *a*	Chl *a*	Chl *a*	Chl *a*	Chl *a*	Chl *a*	Chl *a*	Chl *a*	Chl *a*	Chl *a*	Chl *a*	Chl *a*	Chl *a*	Chl *a*	Chl *a*	Chl *a*	Chl *a*	Chl *a*	Chl *a*	Chl *a*	Chl *a*	Chl *a*
610	408	Chl *c*	Chl *c*	Chl *a*	Chl *a*	Chl *a*	Chl *c*	Chl *c*	Chl *c*	Chl *c*	Chl *c*	Chl *c*	Chl *c*	Chl *c*	Chl *c*	Chl *c*	Chl *c*	Chl *c*	Chl *c*	Chl *c*	Chl *c*	Chl *c*	Chl *c*	Chl *c*	Chl *c*
611	409		Chl *a*	Chl *a*	Chl *a*	Chl *a*	Chl *a*	Chl *a*	Chl *a*	Chl *a*	Chl *a*	Chl *a*	Chl *a*	Chl *a*	Chl *a*	Chl *a*	Chl *a*	Chl *a*	Chl *a*	Chl *a*	Chl *a*	Chl *a*	Chl *a*	Chl *a*	Chl *a*
606	410	Chl *a*			Chl *a*		Chl *a*			Chl *a*	Chl *a*			Chl *a*								Chl *a*		Chl *a*	Chl *a*
612	411				Chl *a*		Chl *a*	Chl *a*		Chl *a*	Chl *a*														
	412				Chl *a*	Chl *a*	Chl *a*				Chl *a*						Chl *a*			Chl *a*					
	413								Chl *a*								Chl *a*			Chl *a*					
	414				Chl *c*																				
	415				Chl *c*																				
	416				Chl *a*											Chl *c*	Chl *c*	Chl *a*			Chl *c*	Chl *a*	Chl *c*	Chl *a*	Chl *a*
	417			Chl *a*																					
	418														Chl *c*										
	419																Chl *c*								
	420																		Chl *a*						
	421																					Chl *a*		Chl *a*	Chl *a*
	422															Chl *a*		Chl *a*			Chl *a*	Chl *a*	Chl *a*	Chl *a*	Chl *a*
	423															Chl *a*									
615	301		Fx	Fx	Fx	Fx	Ddx	Ddx	Fx	Fx	Fx		Fx	Fx		Fx	Fx	Fx	Fx	Fx	Fx	Fx	Fx	Fx	Fx
616	302	Fx	Fx	Fx	Fx	Ddx	Fx	Fx	Fx	Fx	Fx		Fx	Fx	Fx	Fx	Fx	Fx	Fx	Fx	Fx	Fx	Fx	Fx	Fx
614	303	Ddx	Fx	Ddx	Ddx	Ddx	Ddx	Ddx	Ddx	Ddx	Ddx	Ddx	Fx	Ddx	Ddx	Ddx	Ddx	Ddx	Fx	Fx	Ddx	Fx	Ddx	Fx	Fx
	304												Fx												
613	305	Fx	Fx	Fx	Fx	Fx	Ddx	Ddx	Fx	Fx	Ddx	Fx	Fx	Ddx	Fx	Fx	Fx	Ddx	Fx	Fx	Fx	Fx	Fx	Fx	Fx
617	306				Fx	Ddx	Ddx	Ddx	Fx	Fx	Fx	Fx	Fx	Fx	Fx				Fx	Fx		Fx		Fx	Fx
	307				Fx	Fx			Fx			Fx	Fx			Fx	Fx			Fx	Fx	Fx	Fx	Fx	Fx
	308	Ddx			Fx				Fx											Fx				Ddx	Ddx
	309																							Ddx	Ddx
	310								Fx															Fx	Fx

Sites-r: Pigment sites in Lhcr1 of red alga; Sites-d: Pigment sites in FCPI complexes of diatom.

Supplementary Table 3. Comparison of FCPIs between the present and Nagao et al.'s (2020)[10] structure.

Xu et al.	Nagao et al.	Sequences, same or not/number of different residues	Xu et al.-Chls.	Xu et al.-Car.	Nagao et al.-Chls.[10]	Nagao et a.-Car.[10]
FCPI-1, fc13194			6 a, 1 c	2 Ddx, 2 Fx		
FCPI-2, 25468			8 a, 1 c	4 Fx		
FCPI-3, 17531	Fcpa9, lhcr9	Yes	10 a	1 Ddx, 3 Fx	6 a, 4 c	1 Ddx, 3 Fx
FCPI-4, lhcr11	Fcpa8, lhcr8	Yes, 3 a. a.	12 a, 3 c	1 Ddx, 6 Fx	7 a, 7 c	2 Ddx, 5 Fx
FCPI-5, lhcr7	Fcpa7, lhcr7	Yes, 5 a. a.	9 a, 1 c	3 Ddx, 3 Fx	9 a, 2 c	3 Ddx, 3 Fx
FCPI-6, lhcr4	Fcpa6, lhcr6	Yes, 4 a. a.	12 a, 1 c	4 Ddx, 1 Fx	10 a, 4 c	4 Ddx, 1 Fx
FCPI-7, lhcr1	Fcpa5, lhcr5	Yes, 2 a. a. + 7 a. a. insertion in middle + 7 a. a. in C-terminal	10 a, 1 c	4 Ddx, 2 Fx	7 a, 4 c	3 Ddx, 2 Fx
FCPI-8, lhcr10	Fcpa4, lhcr4	Yes, 3 a. a.	8 a, 2 c	1 Ddx, 7 Fx	7 a, 3 c	6 Ddx, 2 Fx
FCPI-9, 270215	Fcpa3, lhcr3	Yes, 5 a. a.	10 a, 1 c	1 Ddx, 4 Fx	8 a, 3 c	3 Ddx, 2 Fx
FCPI-10, tplhcr14	Fcpa2, lhcr2	No	12a, 1 c	2 Ddx, 3 Fx	10 a, 3 c	3 Ddx, 3 Fx
FCPI-11, lhcr3	Fcpa1, lhcr1	Yes, 3 a. a.	9 a, 1 c	1 Ddx, 3 Fx	6 a, 2 c	1 Ddx, 2 Fx
FCPI-12, lhcf4			7 a, 2 c	7 Fx		
FCPI-13, lhcA	Fcpa10, lhcr10	Yes, 4 a. a.	9 a, 1 c	2 Ddx, 3 Fx	7 a, 3 c	2 Ddx, 3 Fx
FCPI-14, FCP2	Fcpa11, lhcq1	Yes, 2 a. a.	8 a, 2 c	1 Ddx, 3 Fx	5 a, 4 c	1 Ddx, 3 Fx
FCPI-15, 270221	Fcpa12, lhcq2	No, 40 a. a.	10 a, 2 c	1 Ddx, 4 Fx	8 a, 4 c	2 Ddx, 3 Fx
FCPI-16, 6139	Fcpa13, lhcq3	Yes, 3 a. a.	10 a, 3 c	1 Ddx, 4 Fx	6 a, 6 c	1 Ddx, 2 Fx
FCPI-17, ptlhcf4			1 0a, 1 c	2 Ddx, 2 Fx		
FCPI-18, ptlhcf4			8 a, 2 c	5 Fx		
FCPI-19, 37296			10 a, 1 c	6 Fx		
FCPI-20, 270221			9 a, 2 c	1 Ddx, 4 Fx		
FCPI-21, 10219			12 a, 1 c	6 Fx		
FCPI-22, 270221	Fcpa16, lhcq6	Yes	9 a, 2 c	1 Ddx, 4 Fx	9 a, 2 c	1 Ddx, 2 Fx
FCPI-23, 10219	Fcpa15, lhcq5	Yes, 3 a. a. + 3 a. a. in the C-terminal	12 a, 1 c	2 Ddx, 7 Fx	13 a	2 Ddx, 7 Fx
FCPI-24, 270092	Fcpa14, lhcq4	Yes, 3 a. a.	12 a, 1 c	2 Ddx, 7 Fx	10 a, 3 c	7 Fx

FCPIs whose sequences were different between the two structures are highlighted in red; Chls that were differently assigned in the two structures are highlighted in blue.

Supplementary References

1. Pi, X. et al. Unique organization of photosystem I-light-harvesting supercomplex revealed by cryo-EM from a red alga. *Proc. Nat.l Acad. Sci. U. S. A.* **115,** 4423-4428 (2018).
2. Qin, X., Suga, M., Kuang, T., & Shen J.-R. Photosynthesis. Structural basis for energy transfer pathways in the plant PSI-LHCI supercomplex. *Science* **348,** 989-995 (2015).
3. Wang, W. et al. Structural basis for blue-green light harvesting and energy dissipation in diatoms. *Science* **363,** eaav0365 (2019).
4. Kumar, S., Stecher, G., Li, M., Knyaz, C. & Tamura, K. MEGA X: Molecular Evolutionary Genetics Analysis across computing platforms. *Mol. Biol. Evol.* **35,** 1547-1549 (2018).
5. Dereeper, A. et al. Phylogeny.fr: robust phylogenetic analysis for the non-specialist. *Nucleic Acids Res.* **36,** W465-9 (2008).
6. Saitou, N. & Nei, M. The neighbor-joining method: A new method for reconstructing phylogenetic trees. *Mol. Biol. Evol.* **4,** 406-425 (1987).
7. Felsenstein, J. Confidence limits on phylogenies: An approach using the bootstrap. *Evolution* **39,** 783-791 (1985).
8. Zuckerkand, E. & Pauling, L. Evolutionary divergence and convergence in proteins. in Evolving Genes and Proteins (eds. V. Bryson & H.J. Vogel), pp. 97-166. Academic Press, New York (1965).
9. Morariu, V. I., Srinivasan, B. V., Raykar, V. C., Duraiswami, R. & Davis. L. S. Automatic online tuning for fast Gaussian summation. *Adv. Neural Infor. Proc. Syst*. 21 *(NIPS)* 2008).
10. Nagao, R. et al. Structural basis for assembly and function of a diatom photosystem I-light harvesting supercomplex. *Nat. Comm.* **11,** 2481 (2020).
